# Innate immune responses yield tissue-specific bottlenecks that scale with pathogen dose

**DOI:** 10.1073/pnas.2309151120

**Published:** 2023-09-05

**Authors:** Karthik Hullahalli, Katherine G. Dailey, Matthew K. Waldor

**Affiliations:** ^a^Department of Microbiology, Harvard Medical School, Boston, MA 02115; ^b^Division of Infectious Disease, Brigham & Women’s Hospital, Boston, MA 02115; ^c^HHMI, Boston, MA 02115

**Keywords:** bottlenecks, dose–response, systemic infection, innate immunity

## Abstract

To cause infection, pathogens must overcome bottlenecks imposed by the host immune system. These bottlenecks restrict the inoculum and largely determine whether pathogen exposure results in disease. Infection bottlenecks therefore quantify the effectiveness of immune barriers. Here, using a model of *Escherichia coli* systemic infection, we identify bottlenecks that tighten or widen with higher inoculum sizes, revealing that the efficacy of innate immune responses can increase or decrease with pathogen dose. We term this concept “dose scaling”. During *E. coli* systemic infection, dose scaling is tissue specific, dependent on the lipopolysaccharide (LPS) receptor TLR4, and can be recapitulated by mimicking high doses with killed bacteria. Scaling therefore depends on sensing of pathogen molecules rather than interactions between the host and live bacteria. We propose that dose scaling quantitatively links innate immunity with infection bottlenecks and is a valuable framework for understanding how the inoculum size governs the outcome of pathogen exposure.

The COVID-19 pandemic has catalyzed renewed interest in the long-standing question of how the pathogen inoculum size impacts subsequent infection (1, 2). A critical parameter that determines whether a given pathogen dose can establish infection is the bottleneck, which represents host processes that eliminate inoculated microorganisms (PMID: 31992714 and PMID: 36757366) (3–8). Organisms that survive bottlenecks and give rise to the population at an infection site are known as the founding population (FP) (9). FP cannot be measured by enumerating colony-forming units (CFU) alone since total burden is governed by both infection bottlenecks and pathogen replication.

We developed Sequence Tag Based Analysis of Microbial Populations in R (STAMPR), a methodology that quantifies FP with barcoded bacteria (10). The relationship between FP and dose measures the bottleneck (3, 4). The few studies that have measured bottlenecks with barcoded bacteria have focused on enteric infections and found that bottlenecks restrict fixed fractions of the inoculum, rather than fixed numbers (3, 4). Thus, barriers to enteric colonization, such as stomach acid and the microbiota, have similar fractional efficacies at low or high doses. However, in other infection models, bottlenecks may constrict or widen in response to changes in dose. For example, positive feedback in the immune system may tighten bottlenecks at higher doses, or bottlenecks may widen at higher doses if immune effectors are overwhelmed.

We recently profiled the dynamics of systemic *Escherichia coli* infection in mice (11). Following intravenous (IV) inoculation, multiple interconnected components of the innate immune system, including the production of proinflammatory cytokines and infiltration of immune cells to tissues, are rapidly triggered in a manner dependent on TLR4. These factors impose bottlenecks and govern infection establishment; FP explicitly quantifies the collective efficacy of these innate immune constituents at a single dose. The dose–FP relationship (i.e., the bottleneck) thus quantifies innate immune potency across all doses. Here, we leveraged STAMPR to define the broader role of TLR4 in modulating the dynamics of *E. coli* systemic infection and provide a conceptual link between dose, bottlenecks, and innate immunity.

## Results and Discussion

### Dose-FP Curves Reveal Tissue-Specific Potency of Innate Immunity.

**Fig. 1.**
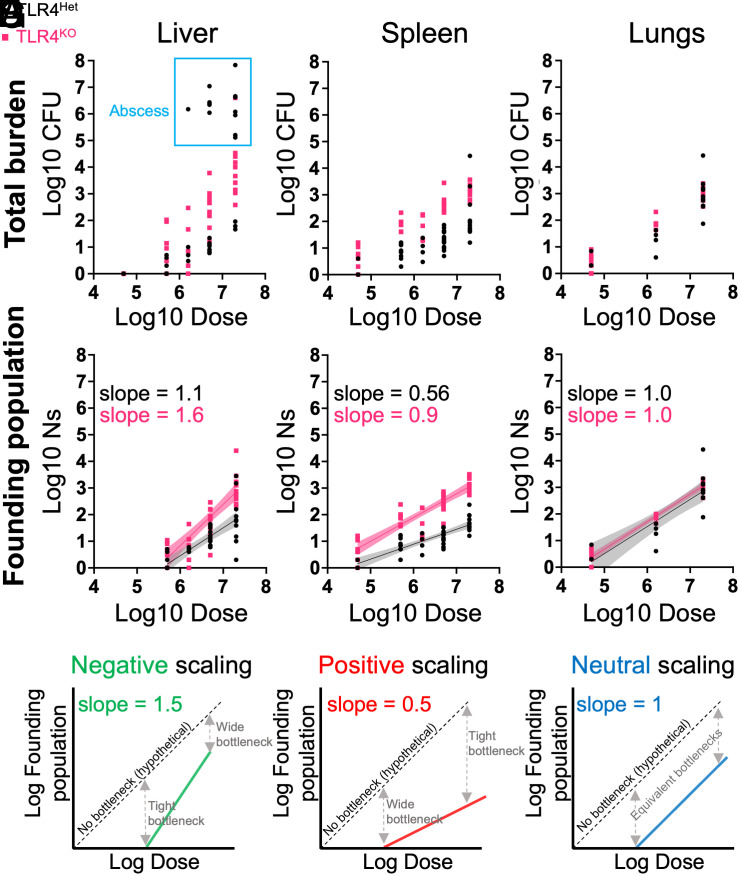
Bottleneck–dose–response analysis for *E. coli* systemic infection. (*A* and *B*) CFU (*A*) and FP (Ns) (*B*) are shown for the liver as a function of dose. Each point represents an animal, and the blue box indicates animals with abscesses. *C*–*F* are identical to *A* and *B* but represent spleen and lung CFU (*C* and *E*) and FP (*D* and *F*). Best fit lines from linear regression in FP plots are shown with 95% confidence bands. (*G*) Dose–FP plots for different scaling patterns. Dotted lines represent a 0% bottleneck. With slopes greater than one, fractionally more bacteria survive to become founders at higher doses; innate immune responses are less effective at higher inoculum sizes and therefore “negatively scale” with dose. With slopes less than one, the immune response is “positively scaled” since fractionally fewer bacteria survive at higher doses. With a slope equal to 1, a fixed fraction of the inoculum survives host bottlenecks.

TLR4 knockout mice (TLR4^KO^) mice and heterozygous littermates (TLR4^Het^) were intravenously inoculated with varying doses of barcoded *E. coli*. Five days postinfection (dpi), the lungs, liver, and spleen were harvested for bacterial enumeration and STAMPR analysis. All organs exhibited dose-dependent increases in CFU. At higher doses, abscesses appeared in the livers of TLR4^Het^ animals, but not in TLR4^KO^ animals (Fig. 1*A*, blue box). Consistent with our companion study (12), abscesses result from bacterial replication (low FP and high CFU). TLR4^KO^ mice are resistant to abscess formation (Fig. 1*A*, outside blue box) but have higher FP (Fig. 1*B*), indicating that in the absence of TLR4, more bacteria from the inoculum survive infection bottlenecks. In the spleen, TLR4^KO^ animals also had higher burdens than TLR4^Het^ animals (Fig. 1*C*). FPs were also higher in TLR4^KO^ spleens, revealing that TLR4 controls splenic bottlenecks (Fig. 1*D*). In contrast to both the liver and spleen, neither bacterial burden nor FP was influenced by TLR4 in the lungs (Fig. 1 *E* and *F*).

Bottlenecks are quantified by the relationship between FP and dose (3, 4). Bottlenecks with a slope of 1 on dose–FP curves indicate that numerically more bacteria are eliminated at higher doses, but the fraction killed (and fraction surviving) is constant. TLR4^KO^ animals had higher dose–FP slopes compared to littermate TLR4^Het^ animals in the spleen [0.9 ± 0.07 (SE) vs. 0.56 ± 0.06, Fig. 1*D*] and liver (1.6 ± 0.17 vs. 1.1 ± 0.14, Fig. 1*B*). Although TLR4 influences the slopes in both organs by a similar magnitude, the specific numerical values reveal tissue-specific differences in the potency of innate immunity. The slope <1 in the TLR4^Het^ spleen is an example of “positive scaling” (Fig. 1*G*), where innate immunity is more effective at higher inoculum sizes; fractionally fewer bacteria survive to become founders at larger doses (fractionally more are killed). Positive scaling in the spleen is dependent on TLR4 since in its absence, the slope increases to ~1 (“neutral scaling” Fig. 1*G*). Neutral scaling was observed in the livers of TLR4^Het^ animals. In the absence of TLR4 in the liver, the slope increased to >1; fractionally more bacteria survive at higher doses (fractionally fewer are killed). Thus, in the absence of TLR4, the hepatic innate immune response is fractionally less effective at larger doses, which we term “negative scaling”. In contrast to the liver and spleen, we observed neutral scaling in the lung independent of TLR4. These results reveal that in addition to controlling tissue-specific immune responses, TLR4 governs the extent to which the efficacy of these responses quantitatively scales with inoculum size; mice lacking TLR4 have disproportionately reduced potency of innate immune responses at higher doses. Notably, TLR4 not only controls pathogen burdens but also their diversity, which may be important for longer-term population-level outcomes such as transmission of drug resistance or the emergence of hypervirulent pathogen lineages.

### Dose Scaling Does Not Require Live Bacteria.

**Fig. 2.**
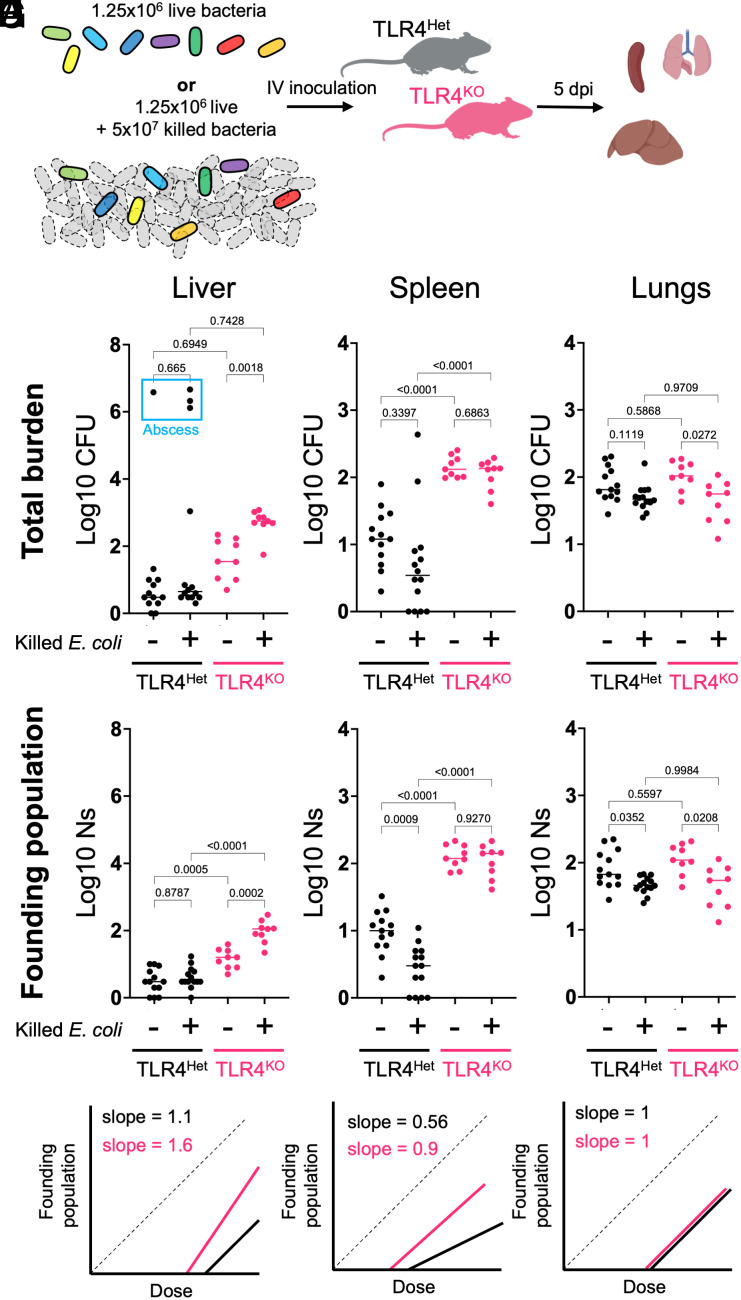
Tissue- and TLR4-dependent responses to spike-in of killed bacteria. (*A*) Barcoded live bacteria or the same quantity of live cells plus 40-fold excess of formalin-fixed bacteria were IV inoculated into TLR4^Het^ or TLR4^KO^ littermates. (*B* and *C*) CFU (*B*) and FP (Ns) (*C*) of the liver are shown (lines represent medians). The blue box indicates animals with abscesses. (*D*–*G*) are identical to *B* and *C* but represent spleen and lung CFU (*D* and *F*) and FP (*E* and *G*). (*H*) Curves from Fig. 1 (*Left* to *Right*: liver, spleen, and lung) are schematized for reference.

We hypothesized that dose scaling is separable from the true number of inoculated organisms and is instead dependent on how the innate immune system senses and “interprets” the inoculum size. To test this idea, we spiked killed bacteria into a live inoculum to simulate some of the immune-stimulatory aspects of high doses. TLR4^Het^ and TLR4^KO^ animals were inoculated with 1.25 × 10^6^ CFU live bacteria or the same quantity of live cells plus 5 × 10^7^ formalin-killed *E. coli* (Fig. 2*A*). Both groups received identical amounts of live bacteria (mean log10 dose ± St.dev, control: 6.03 ± 0.14, spike-in: 6.13 ± 0.10). CFU (Fig. 2 *B*, *D*, and *F*) and FP (Fig. 2 *C*, *E*, and *G*) were calculated at 5 dpi.

The potency of splenic immune responses was greater at higher doses (positive scaling) in a manner dependent on TLR4 (Fig. 1*D*). Consistent with increased innate immune efficacy at higher doses, spike-in of killed bacteria yielded threefold fewer live founders in TLR4^Het^ spleens (Fig. 2*E*), and the decrease in FP was not observed in the absence of TLR4. In contrast, the livers of TLR4^KO^ animals were scaled negatively (Fig. 1*B*). With spiked killed cells, TLR4^KO^ livers had significantly greater CFU and fivefold more founders than control animals without spiked-in killed cells (Fig. 2*C*). No changes in CFU or FP were seen with spiked-in cells in the livers of TLR4^Het^ animals, consistent with neutral scaling (Fig. 2*C*). Therefore, positive scaling in the spleen and negative scaling in the liver do not require live bacteria.

The magnitude of changes in FP in response to killed cells is close to predictions from dose–FP curves (Fig. 1). By multiplying the fractional bottleneck (FP/Dose) at a 2 × 10^7^ dose with an actual dose of 1.25 × 10^6^, the TLR4^Het^ spleen and TLR4^KO^ liver are predicted to have threefold fewer and fourfold greater founders, respectively, relative to control animals without spiked-in cells. The effect of killed cells on CFU in the spleen is not statistically significant despite a significant difference in FP since two mice had stochastic bacterial replication (Fig. 2*D*). However, the livers of TLR4^KO^ mice had significantly greater CFU after spike-in (Fig. 2*B*). Thus, when bacteria can replicate substantially, FP rather than CFU is a more faithful measure of scaling since it is not confounded by bacterial expansion. Although the lungs exhibited neutral scaling, we observed a minor but statistically significant decrease in CFU and FP following spike-in (Fig. 2 *F* and *G*).

These data show that dose scaling can be recapitulated without increasing the inoculum size. Furthermore, the two seemingly opposite effects of spiking in killed cells (higher FP in TLR4^KO^ livers but lower FP in the TLR4^Het^ spleen) result from a similar conceptual basis in dose scaling. The dependence of the effects of killed cell spike-in on TLR4 suggests that dose scaling results from quantitative changes in LPS-induced immune responses, rather than the actual pathogen dose. The increase in liver FP following spike-in only in the absence of TLR4 suggests that pathogen molecules other than LPS yield negative scaling patterns. We speculate that the host pathways engaged by these other molecules are more easily exhausted in the absence of TLR4. Other pathogen and host factors that control immune responses, such as TLR5 (flagellin) or Nod2 (peptidoglycan), may benefit from analysis with the dose–FP paradigm to decipher how innate immunity scales with dose to regulate infection outcome.

## Concluding Remarks

We describe the concept of dose scaling, which relates changes in inoculum size with quantitative changes in infection bottlenecks and the efficacy of innate immune responses. We show that different pathogen doses can yield tighter or wider bottlenecks in a tissue-specific manner. Since scaling can in part be recapitulated with killed organisms, leveraging scaling may represent a framework to identify therapeutics that tighten infection bottlenecks. We hypothesize that positive and negative scaling arise from differences in the rate of induction of individual components of the innate immune response as a function of dose. These differences are likely influenced by the underlying immune cell composition of different tissues as well as tissue-specific gene expression patterns. Further studies of dose scaling will provide critical contextualization for natural infections, where inoculum sizes are not controlled.

## Materials and Methods

Ns calculations were performed as previously described (4). Note that all liver CFU calculations are reported for ¼ of the liver to accurately compare Ns and CFU. Details of animal experiments and STAMPR analysis are provided in *SI Appendix, Materials and Methods*.

## Supplementary Material

Appendix 01 (PDF)

## Data Availability

All study data are included in the article and/or *SI Appendix*. STAMPR code is available at https://github.com/hullahalli/stampr_rtisan (13).

## References

[1] L. G. Rubin, Bacterial colonization and infection resulting from multiplication of a single organism. Rev. Infect Dis. **9**, 488–493 (1987). 3299635 10.1093/clinids/9.3.488

[2] E. E. Bendall , Rapid transmission and tight bottlenecks constrain the evolution of highly transmissible SARS-CoV-2 variants. Nat. Commun. **14**, 1–7 (2023). 36650162 10.1038/s41467-023-36001-5 PMC9844183

[3] A. N. Gillman, A. Mahmutovic, P. Abel zur Wiesch, S. Abel, The Infectious Dose Shapes Vibrio cholerae Within-Host Dynamics. mSystems **6**, e00659–21 (2021). 34874769 10.1128/mSystems.00659-21 PMC8651084

[4] I. W. Campbell, K. Hullahalli, J. R. Turner, M. K. Waldor, Quantitative dose-response analysis untangles host bottlenecks to enteric infection. Nat. Commun. **14**, 1–13 (2023). 36709326 10.1038/s41467-023-36162-3 PMC9884216

[5] E. J. G. Pollitt, P. T. Szkuta, N. Burns, S. J. Foster, Staphylococcus aureus infection dynamics. PLoS Pathog. **14**, e1007112. (2018). 29902272 10.1371/journal.ppat.1007112 PMC6019756

[6] T. Zhang , Deciphering the landscape of host barriers to Listeria monocytogenes infection. Proc. Natl. Acad. Sci. U.S.A. **114**, 6334–6339 (2017). 28559314 10.1073/pnas.1702077114 PMC5474794

[7] K. E. R. Bachta , Systemic infection facilitates transmission of Pseudomonas aeruginosa in mice. Nat. Commun. **11**, 543 (2020). 10.1038/s41467-020-14363-4 PMC6987207 31992714

[8] D. Hoces , Fitness advantage of *Bacteroides thetaiotaomicron* capsular polysaccharide in the mouse gut depends on the resident microbiota. Elife. **12**, e81212 (2023). 36757366 10.7554/eLife.81212 PMC10014078

[9] S. Abel, P. Abel zur Wiesch, B. M. Davis, M. K. Waldor, Analysis of Bottlenecks in Experimental Models of Infection. PLoS Pathog. **11**, e1004823. (2015). 26066486 10.1371/journal.ppat.1004823 PMC4465827

[10] K. Hullahalli, J. R. Pritchard, M. K. Waldor, Refined Quantification of Infection Bottlenecks and Pathogen Dissemination with STAMPR. mSystems **6**, e00887–21 (2021). 34402636 10.1128/mSystems.00887-21 PMC8407386

[11] K. Hullahalli, M. K. Waldor, Pathogen clonal expansion underlies multiorgan dissemination and organ-specific outcomes during murine systemic infection. Elife **10** (2021). 10.7554/eLife.70910 PMC8545400 34636322

[12] K. Hullahalli , Genetic and immune determinants of E. coli liver abscess formation. bioRxiv **543319** (2023). 10.1073/pnas.2310053120 PMC10743367 38096412

[13] K. Hullahalli, K. G. Dailey, M. K. Waldor, DoseScalingTLR4. stampr_rtisan. https://github.com/hullahalli/stampr_rtisan. Deposited 17 August 2023.

