# Supplementary material for: Innate immune responses yield tissue-specific bottlenecks that scale with pathogen dose
Source: Proc Natl Acad Sci U S A. 2023 Sep 5;120(37):e2309151120. doi: 10.1073/pnas.2309151120 (PMC10500177; doi:10.1073/pnas.2309151120)
Supplement: Supplementary file 1 — Appendix 01 (PDF) [file pnas.2309151120.sapp.pdf]

Supporting Information

Materials and Methods

Ethics Statement

All animal experiments were conducted in accordance with the recommendations in the Guide for the Care and Use of Laboratory Animals of the National Institutes of Health and the Animal Welfare Act of the United States Department of Agriculture using protocols reviewed and approved by Brigham and Women's Hospital Committee on Animals (Institutional Animal Care and Use Committee protocol number 2016N000416 and Animal Welfare Assurance of Compliance number A4752-01)

Bacterial culture

The *E. coli* strain used in this study was CHS7, derived from strain CFT073 (1). All experiments used a barcoded library of CHS7(2). Bacteria were routinely plated on LB + 50 µg/ml kanamycin agar plates for CFU enumeration.

Animal Experiments

The $TLR4^{KO}/TLR4^{Het}$ colony was established by crossing $TLR4^{KO}$ males (Jackson stock #029015) with C57Bl/6J (Jackson stock #000664) females. $TLR4^{Het}$ female offspring were crossed with $TLR4^{KO}$ males to generate experimental $TLR4^{KO}$ and $TLR4^{Het}$ littermates. Animals were bred in a Biosafety Level 1 helicobacter-free facility at the Brigham and Women's Hospital. At 7-8 weeks of age, animals were transferred to a Biosafety Level 2 room. At 8-10 weeks old, animals (males and females) were inoculated intravenously with the barcoded library of CHS7. The bacterial inoculum was prepared by thawing small frozen aliquots of a master lot of barcoded CHS7 and diluting directly in PBS. To prepare the inoculum containing killed bacteria, frozen aliquots were diluted in 4% paraformaldehyde and incubated at room temperature for 20 minutes. Cells were centrifuged, washed in PBS, and resuspended in 1ml of PBS, after which live bacteria were added. The 100µl inoculum was delivered intravenously via the lateral tail vein with a 27G needle while animals were restrained in a Broome-style restrainer (Plas Labs). A heating pad was used to facilitate dilation of the tail vein. At 5 days post inoculation, mice were euthanized with isoflurane and cervical dislocation, after which the lungs, spleen, and liver were harvested. Prior to harvesting the liver, the bile was aspirated, and the gallbladder was removed. Organs were homogenized by bead beating with two 3.2mm stainless steel beads for two minutes in PBS.

STAMPR analysis

1ml of organ homogenates were plated on 150mm agar plates. Note that liver samples were homogenized in a total of 4ml, but only 1ml was plated for STAMPR analysis. To accurately compare CFU and FP values, all liver CFU values reported in this study are for ¼ of the liver. Colonies were picked or lawns were scraped into PBS + 25% glycerol and stored at -80º C.

To amplify the barcode region, cell suspensions were diluted ~1:50 in water, or up to 1:10 if cell density was low (i.e., few CFU in the organ). Diluted suspensions were boiled (95ºC for 15 min) and 3µl was used for PCR as previously described, with minor modifications (3). PCR reactions were performed with 2x OneTaq HotStart DNA Polymerase master mix (New England Biolabs), using 6 µl of forward and reverse primer (1µM each). The forward primers were designed such that the first nucleotides to be sequenced were a region of variable length to facilitate optimal color balancing. These variability regions also contained sequence diversity for multiplexing and demultiplexing. Reverse primers contained i7 indexes from the TruSeq DNA PCR Free kit (Illumina). PCR reactions were run on agarose gels to verify amplification, pooled, and then purified using the GeneJet PCR purification kit. Amplicons were quantified with Qubit and sequenced as 1 x 78 nt reads on MiSeq or NextSeq 1000 instruments (Illumina)

Reverse primer demultiplexing with i7 was performed on BaseSpace (Illumina), and forward primer demultiplexing was performed using custom R scripts. Demultiplexed reads (.fastq files) were trimmed and mapped to a previously defined list of reference barcodes using CLC genomics workbench. Read count tables, where columns specify the sample and rows specify the barcode, were exported as .csv files and used for subsequent analysis. For samples sequenced on a NextSeq 1000, we included an additional index hopping correction. For each sample n_0, we identified every other sample n_i that was separated by only one index. Barcode counts were combined for all n_i samples, multiplicatively scaled, and subtracted from sample n_0. No more than 3% of reads were removed from any sample.

Founding population size was approximated by calculation of Ns, which is defined as the number of times the input library must be sampled from a multinomial distribution to observe a given number of barcodes. Ns calculation was performed as previously described(3, 4) . Briefly, read count tables were first converted to frequencies. To identify noise thresholds, we first identified large gaps in barcode frequency between sorted barcodes (greater than 10-fold) as well as breaks that delineate sub-populations as defined by the STAMPR algorithm (described extensively in (5)). All barcodes below the frequency threshold were defined as noise and set to 0 reads. Ns was calculated by first resampling the input library to the read depth of the output sample. The resulting distribution was then iteratively resampled from 1 to ~10,000 times. A sampling size of ~10,000 is sufficient to detect all ~1000 barcodes in the library and defines the maximum resolution of Ns. A “reference resample curve” was generated, where the x-axis is the number of times sampled (Ns) and the y-axis is the number of barcodes detected. The number of barcodes in each sample after noise thresholding was used as input for inverse interpolation from the reference resample curve to calculate Ns.

Statistics

Linear regression in Figure 1 was performed in GraphPad Prism. This study assumes that curves are linear, but it is possible that with a substantially larger number of replicates a nonlinear fit would be a more appropriate approximation of the dose-FP trend (6). The 5 x 10^4 dose was excluded from linear regression analysis in the liver, since 7 animals had 0 CFU and 4 animals had 1 CFU. Statistical significance in Figure 2 was assessed using Brown-Forsythe and Welch ANOVA tests followed by Dunnett’s T3 multiple comparisons test (GraphPad Prism).

References

1. Welch RA, Burland V, Plunkett G, Redford P, Roesch P, Rasko D, Buckles EL, Liou S-R, Boutin A, Hackett J, Stroud D, Mayhew GF, Rose DJ, Zhou S, Schwartz DC, Perna NT, Mobley HLT, Donnenberg MS, Blattner FR. 2002. Extensive mosaic structure revealed by the complete genome sequence of uropathogenic Escherichia coli. Proc Natl Acad Sci U S A 99:17020–4.
2. Hullahalli K, Waldor MK. 2021. Pathogen clonal expansion underlies multiorgan dissemination and organ-specific outcomes during murine systemic infection. Elife 10:e70910.
3. Campbell IW, Hullahalli K, Turner JR, Waldor MK. 2023. Quantitative dose-response analysis untangles host bottlenecks to enteric infection. Nature Communications 2023 14:1 14:1–13.
4. Fakoya B, Hullahalli K, Rubin DHF, Leitner DR, Chilengi R, Sack DA, Waldor MK. 2022. Nontoxigenic Vibrio cholerae Challenge Strains for Evaluating Vaccine Efficacy and Inferring Mechanisms of Protection. mBio 13:e0053922.
5. Hullahalli K, Pritchard JR, Waldor MK. 2021. Refined Quantification of Infection Bottlenecks and Pathogen Dissemination with STAMPR. mSystems 6:e00887-21.
6. Gillman AN, Mahmutovic A, Abel zur Wiesch P, Abel S. 2021. The Infectious Dose Shapes Vibrio cholerae Within-Host Dynamics. mSystems 6:e00659-21.
